# Community-based lifestyle interventions and psychological well-being among rural older adults: Evidence from Taiwan’s green care program

**DOI:** 10.1016/j.jarlif.2026.100074

**Published:** 2026-05-28

**Authors:** Kai-Lin Liang, Yi-Chun Hung, Chia-Chen Hung, Chih-Ying Lin

**Affiliations:** a Aging Health and Long-Term Care Management for Indigenous Undergraduate Program, National Chi Nan University, Taiwan. No 1, University Rd., Puli Township, Nantou County, Nantou, Taiwan; b Institute of Population Health Sciences, National Health Research Institutes, Miaoli, Taiwan; c Kaohsiung Medical University Gangshan Hospital, Kaohsiung, Taiwan

**Keywords:** Green care, Lifestyle intervention, Intrinsic capacity, ICOPE, Social prescribing, Well-being

## Abstract

**Background:**

Community-based lifestyle interventions, including nature-based and Green Care activities, are associated with improved psychological well-being among older adults. However, these programs are often delivered as standardized interventions without systematic alignment with individual functional capacity or psychosocial needs. The World Health Organization’s Integrated Care for Older People (ICOPE) framework is a person-centred, integrated care approach spanning assessment, care planning, and community linkage, offering a multidimensional structure for assessing intrinsic capacity and guiding personalized participation.

**Objective:**

This study examined the association between participation in community-based lifestyle intervention domains and psychological well-being among rural older adults and evaluated the potential of the ICOPE framework as a classification structure for precision community participation.

**Methods:**

A cross-sectional study was conducted among community-dwelling older adults in rural Taiwan. Psychological well-being was assessed using the WHO-5 Well-Being Index. Participation in lifestyle intervention domains was analyzed, and cluster analysis was used to identify heterogeneous participation profiles.

**Results:**

Distinct participation clusters with varying well-being profiles were identified. Higher engagement in community-based and nature-based interventions was associated with significantly higher psychological well-being.

**Conclusion:**

Community-based lifestyle and Green Care interventions were associated with psychological well-being outcomes among rural older adults. As a person-centred, integrated care framework, ICOPE offers a promising conceptual structure for organizing community participation programs and supporting precision social prescribing, though direct testing of ICOPE-guided intervention matching was beyond the scope of this study. Personalized community-based health promotion.

## Highlights

• Green Care participation is associated with psychological well-being in rural older adults.
• Cluster analysis reveals heterogeneous participation and well-being patterns.
• ICOPE’s integrated care framework may support classification of community participation programs.
• ICOPE offers a plausible foundation for precision social prescribing in aging populations.
• Nature-based interventions promote sustainable healthy aging.

## 1 Introduction

Population aging presents profound challenges for healthcare systems and community services worldwide, particularly in maintaining psychological well-being, functional ability, and quality of life among older adults. Psychological well-being is a critical component of healthy aging, as it is strongly associated with functional independence, resilience, and reduced risk of adverse health outcomes. Lifestyle interventions, including physical activity, psychosocial engagement, and community participation, have been widely recognized as effective strategies for enhancing psychological well-being in older populations [[1], [2], [3]]. These interventions improve well-being through mechanisms such as enhanced self-efficacy, social engagement, and cognitive and emotional regulation [2]. Community-based health promotion programs, in particular, have demonstrated effectiveness in improving psychological health, promoting health behaviors, and enhancing overall well-being, especially when interventions are delivered within socially supportive environments [4,5].

Social participation plays a central role in promoting psychological well-being and healthy aging. Engagement in community activities has been associated with reduced depressive symptoms, improved emotional health, and enhanced social connectedness [6,7]. In response to the growing recognition of social determinants of health, social prescribing has emerged as an innovative care model that connects individuals with community-based activities to improve health and well-being [[8], [9]]. Social prescribing interventions, including participation in gardening, cultural programs, and volunteer activities, have demonstrated effectiveness in reducing social isolation and improving psychological well-being among older adults [10]. However, despite the demonstrated benefits of social participation and lifestyle interventions, existing programs are often delivered as standardized interventions without systematic alignment with individual functional capacity or psychosocial needs [[11], [12], [13]].

Among lifestyle interventions, nature-based interventions and Green Care programs have gained increasing recognition as effective approaches for promoting psychological well-being and healthy aging. For the purposes of this study, “Green Care” refers to the Rural Community Green Care Program—a structured, multidimensional lifestyle intervention implemented by Taiwan’s Ministry of Agriculture—which integrates nature-based and lifestyle activities for community-dwelling older adults. This program represents one application within the broader family of nature-based interventions (NBIs), which encompasses horticultural therapy, nature-based physical activities, community gardening, and related approaches utilizing natural environments for therapeutic and preventive purposes. Green Care interventions of this kind have been shown to be associated with reduced symptoms of depression and anxiety, and with improvements in life satisfaction, emotional regulation, and overall well-being [[14], [15], [16], [17]]. These interventions are understood to promote psychological well-being through multiple pathways, including exposure to restorative natural environments, meaningful engagement in purposeful activities, and opportunities for social interaction and peer support [18,19]. The psychological benefits of nature-based interventions are further supported by theoretical frameworks such as Attention Restoration Theory and Stress Reduction Theory, which highlight the restorative and stress-reducing effects of natural environments [20]. In addition to their psychological benefits, community-based Green Care programs have demonstrated feasibility, accessibility, and sustainability, making them promising interventions for promoting healthy aging, particularly in community and rural settings [21].

Despite the growing evidence supporting Green Care and community-based lifestyle interventions, a key challenge remains in systematically identifying individual functional needs and matching older adults with appropriate interventions. The World Health Organization’s Integrated Care for Older People (ICOPE) framework was developed to address this challenge by providing a comprehensive approach to assessing intrinsic capacity across multiple domains, including cognition, mobility, vitality, psychological health, and sensory function [22,23]. Intrinsic capacity is a central determinant of functional ability and healthy aging, and early identification of domain-specific decline enables targeted preventive interventions [[24], [25]]. It is important to clarify that ICOPE is not merely a screening instrument; rather, WHO presents it as a person-centred, integrated health and social care approach encompassing assessment, individualized care planning, and linkage to community services. This integrated care framework, grounded in the WHO Healthy Ageing framework, offers substantial potential for organizing, classifying, and guiding community-based lifestyle interventions at the population level.

Importantly, the multidimensional structure of ICOPE aligns closely with the diverse functional targets of Green Care and lifestyle interventions. Nature-based interventions, physical activity programs, and social participation activities correspond to distinct intrinsic capacity domains, including psychological health, mobility, and vitality. Integrating the ICOPE framework with Green Care and community-based lifestyle interventions provides an opportunity to move beyond generalized participation models toward precision social prescribing, in which interventions are systematically matched to individual functional needs. Such an approach enables the development of heterogeneous participation pathways that simultaneously promote social engagement, enhance intrinsic capacity, and improve psychological well-being.

Furthermore, older adult populations exhibit substantial heterogeneity in functional ability, psychological well-being, and social participation patterns [26,27]. However, most community participation programs currently provide uniform intervention content without considering individual intrinsic capacity profiles. This gap highlights the need for integrated frameworks that combine functional assessment with targeted intervention delivery. The integration of ICOPE with Green Care and community-based lifestyle interventions offers a promising model for bridging this gap by providing a structured framework for both assessing intrinsic capacity and guiding personalized participation and social prescribing.

Therefore, the present study aimed to examine the association between participation in community-based lifestyle intervention domains, including nature-based and Green Care activities, and psychological well-being among rural older adults. In addition, this study sought to identify heterogeneous participation patterns using cluster analysis and to evaluate the potential of the ICOPE framework as a classification structure for organizing community-based lifestyle interventions and guiding precision social prescribing. By integrating intrinsic capacity assessment with Green Care and community participation programs, this study contributes to the development of personalized, community-based intervention models for promoting psychological well-being and healthy aging.

## 2 Methods

### 2.1 Participants and procedure

This study employed a cross-sectional research design using data derived from the 2025 Rural Community Green Care Program, a nationwide community-based initiative sponsored by the Ministry of Agriculture in Taiwan. The program was developed to promote healthy aging among rural community-dwelling older adults through multidimensional lifestyle interventions integrating physical activity, nutrition, cognitive stimulation, sensory health, and social engagement within natural and community settings.

Participants were recruited from rural community care centers located in central Taiwan, including Changhua, Nantou, Yunlin, and Chiayi counties. Eligibility criteria included being aged 65 years or older and actively participating in Green Care program activities. Exclusion criteria comprised cognitive impairment precluding self-report completion or inability to provide informed consent. A total of 1100 participant records were initially collected through routine program health assessments and psychosocial surveys conducted by program staff. One record was excluded due to missing data on the primary outcome variable (WHO-5 Well-being Index), yielding a final analytical sample of 1099 participants (99.9% retention). Missing data were not imputed; analyses were conducted on participants with complete data across all key variables. A simple participant flow diagram is provided in Supplementary Figure 1.

The mean age of participants was 79.83 years (SD = 7.48), ranging from 65 to 100 years. The sample was predominantly female, reflecting the demographic characteristics commonly observed in rural aging populations. Approximately one-quarter of participants lived alone, a factor associated with increased risk of social isolation.

This study was approved by the Institutional Review Board of the National Health Research Institutes (NHRI), Taiwan (IRB No EC1130907-E). All procedures complied with ethical standards for human subjects research.

### 2.2 Green care lifestyle intervention framework

The Rural Community Green Care Program represents a multidimensional lifestyle intervention model grounded in preventive gerontology and lifestyle medicine principles. The program integrates six primary intervention domains aligned with the World Health Organization’s Integrated Care for Older People (ICOPE) framework:

1. Green Exercise: Nature-based physical activities designed to improve mobility, strength, and physical functioning.
2. Green Nutrition: Nutrition education emphasizing healthy dietary practices using locally produced agricultural foods.
3. Green Cognition: Cognitive stimulation activities, including horticultural therapy and structured group engagement, aimed at preserving cognitive function.
4. Vision Health: Vision screening and related health education to support sensory functioning.
5. Hearing Health: Hearing assessments and interventions to enhance communication and reduce sensory-related social isolation.
6. Psychological Support: Group-based emotional support and social interaction activities designed to prevent loneliness and promote psychological well-being.

All six intervention domains were delivered through community care centers by trained health professionals, including public health nurses, nutritionists, occupational therapists, and social workers. Sessions were conducted in group formats (typically 15–20 participants per group) in both indoor community center settings and outdoor natural or agricultural environments. Each domain was delivered at a frequency of approximately 1–2 sessions per week, with individual session durations ranging from 90 to 120 min. Programs operated on a continuous enrollment basis throughout 2025, and participants could attend across multiple domains during the year. Participation was voluntary and self-directed, subject to program availability, rather than formally assigned based on individual assessment. The nature-based elements of the program were operationalized through outdoor settings (e.g., community gardens, agricultural fields, parks) and through horticultural and environmental activities embedded within the Green Exercise and Green Cognition domains in particular. It should be noted that, while the program’s domain structure is conceptually aligned with ICOPE’s intrinsic capacity domains, formal ICOPE-guided assessment and referral matching were not implemented in this study. The ICOPE framework is therefore applied here as a conceptual and classificatory lens rather than a description of clinical practice.

### 2.3 Measures

#### 2.3.1 Psychological well-being

Psychological well-being was assessed using the World Health Organization Five Well-being Index (WHO-5), a widely validated instrument measuring subjective psychological well-being. The WHO-5 consists of five items rated on a 6-point Likert scale ranging from 0 (at no time) to 5 (all of the time), with higher scores indicating better psychological well-being.

#### 2.3.2 Social participation

Social participation was measured using a 12-item Social Participation Scale assessing engagement in community, recreational, and social activities. Higher scores indicate greater levels of social participation.

#### 2.3.3 Social connection and social isolation risk

Social connection was assessed using an 8-item Social Connection Scale measuring perceived interpersonal connection and social support. Consistent with prior research, participants scoring below the sample median were classified as being at higher risk of social isolation.

#### 2.3.4 Intervention intensity (Lifestyle intervention exposure)

Intervention intensity was operationalized as the cumulative annual hours of participation in each Green Care lifestyle intervention domain, including green exercise, nutrition education, cognitive stimulation, vision health, hearing health, and psychological support activities.

Participation data were obtained from standardized attendance records maintained by trained community program coordinators as part of routine program administration. These records systematically documented individual attendance for each intervention session.

For analytical purposes, the total number of participation hours in each intervention domain was treated as continuous exposure variables. Higher values represented greater engagement in lifestyle intervention activities. This operationalization allowed examination of dose–response relationships between lifestyle intervention engagement and health outcomes.

#### 2.3.5 Covariates

Demographic variables included age, gender, and living status (living alone vs. living with family). These variables were included as covariates due to their established associations with psychological well-being and social isolation in older adult populations.

### 2.4 Statistical analysis

All statistical analyses were conducted using Python 3.10.First, descriptive statistics were calculated to summarize participant characteristics, including means, standard deviations, and score ranges for key variables.Second, multiple linear regression analyses were performed to examine associations between intervention intensity across lifestyle domains and psychological well-being. Intervention hours in each domain were included as independent variables, with WHO-5 well-being score as the dependent variable. Age, gender, and living status were included as covariates.Third, binary logistic regression analyses were conducted to examine associations between intervention intensity and risk of social isolation. Odds ratios (OR) and associated statistical significance were calculated.Finally, K-means cluster analysis was conducted to identify distinct psychosocial and lifestyle profiles among participants. Clustering variables included psychological well-being, social participation, social connection, and age. All variables were standardized prior to clustering. The optimal number of clusters was determined based on the elbow method and interpretability criteria.

## 3 Results

### 3.1 Participant characteristics

A total of 1099 rural community-dwelling older adults were included in the analysis. The mean age of participants was 79.83 years (SD = 7.48), with an age range of 65 to 100 years. The majority of participants were female (74.6%), while 25.4% were male. Approximately 23.4% of participants reported living alone, whereas 76.6% lived with family members.

The mean psychological well-being score, as measured by the WHO-5 Well-being Index, was 11.08 (SD = 4.23). The mean social participation score was 36.02 (SD = 9.99), and the mean social connection score was 39.26 (SD = 6.94), indicating moderate levels of psychosocial functioning among participants (Table 1).

**Table 1:** Descriptive characteristics of study participants (N = 1099).

Variable	Mean (SD) or n (%)	Range
Age (years)	79.83 (7.48)	65–100
WHO-5 Well-being Score	11.08 (4.23)	5–28
Social Participation Score	36.02 (9.99)	13–60
Social Connection Score	39.26 (6.94)	12–48
Male	279 (25.4%)	—
Female	820 (74.6%)	—
Living alone	257 (23.4%)	—
Living with family	842 (76.6%)	—

### 3.2 Association between lifestyle intervention participation and psychological well-being

Multiple linear regression analysis was conducted to examine the association between participation in Green Care lifestyle intervention domains and psychological well-being (Table 2). The overall regression model was statistically significant, F(9, 1089) = 8.29, p < .001, explaining approximately 6.4% of the variance in well-being scores.

**Table 2:** Multiple linear regression analysis predicting psychological well-being (WHO-5 Score).

Predictor	B	SE	β	t	p
Constant	10.095	1.437	—	7.026	< .001
Green Exercise (hours)	0.002	0.006	.012	0.421	.674
Green Nutrition (hours)	0.002	0.007	.009	0.273	.785
Green Cognition (hours)	–0.005	0.006	–.026	–0.813	.417
Vision Health (hours)	0.019	0.007	.098	2.847	.004
Hearing Health (hours)	–0.017	0.005	–.121	–3.288	.001
Psychological Support (hours)	–0.006	0.007	–.031	–0.970	.332
Age	0.018	0.017	.033	1.023	.307
Male (vs female)	0.422	0.296	.041	1.426	.154
Living with family	0.752	0.308	.072	2.444	.015

Model statistics: R² = .064, Adjusted R² = .056, F(9, 1089) = 8.29, p < .001

Among the intervention domains, greater participation in vision health activities was significantly associated with higher psychological well-being (B = 0.019, p = .004). In contrast, greater participation in hearing health intervention hours was significantly associated with lower well-being scores (B = -0.017, p = .001). Participation in green exercise, nutrition, cognition, and psychological support interventions was not significantly associated with well-being after adjusting for covariates.

Regarding demographic factors, living with family was significantly associated with higher well-being scores compared to living alone (B = 0.752, p = .015). Age and gender were not significant predictors of psychological well-being in the adjusted model.

These findings suggest that specific lifestyle intervention domains, particularly sensory health-related interventions, may be differentially associated with psychological well-being among rural older adults.

### 3.3 Association between lifestyle intervention participation and risk of social isolation

Binary logistic regression analysis was conducted to examine associations between lifestyle intervention participation and risk of social isolation (Table 3). The overall model was statistically significant, χ²(9) = 104.3, p < .001, with a Nagelkerke R² of .118.

**Table 3:** Binary logistic regression analysis predicting risk of social isolation.

Predictor	B	SE	OR	95% CI	p
Constant	3.149	0.760	23.32	5.24–103.66	< .001
Age	–0.038	0.009	0.962	0.945–0.980	< .001
Green Exercise (hours)	–0.009	0.003	0.991	0.985–0.997	.011
Green Nutrition (hours)	0.000	0.003	1.000	0.994–1.006	.934
Green Cognition (hours)	–0.007	0.003	0.993	0.987–0.999	.023
Vision Health (hours)	0.017	0.004	1.017	1.009–1.025	< .001
Hearing Health (hours)	–0.020	0.003	0.981	0.975–0.987	< .001
Psychological Support (hours)	0.008	0.004	1.008	1.001–1.016	.023
Male (vs female)	0.276	0.152	1.318	0.979–1.775	.069
Living with family	–0.163	0.158	0.849	0.623–1.157	.302

Model statistics:

Nagelkerke R² = .118

Model χ²(9) = 104.3, p < .001

Older age was significantly associated with lower risk of social isolation (OR = 0.962, p < .001). Greater participation in green exercise interventions was associated with reduced risk of social isolation (OR = 0.991, p = .011). Similarly, greater participation in cognitive stimulation activities was associated with reduced social isolation risk (OR = 0.993, p = .023). Hearing health intervention participation was also associated with reduced risk (OR = 0.981, p < .001).

In contrast, greater participation in vision health interventions (OR = 1.017, p < .001) and psychological support activities (OR = 1.008, p = .023) was associated with higher likelihood of social isolation risk.

Gender and living status were not statistically significant predictors in the adjusted model.

These findings indicate that engagement in specific lifestyle intervention domains, particularly physical and cognitive activities, was associated with lower risk of social isolation among rural older adults. However, several findings were counterintuitive: vision health participation was positively associated with well-being yet also associated with higher isolation risk, while hearing health participation was negatively associated with well-being yet associated with reduced isolation risk. These patterns should be interpreted cautiously and are discussed in detail in the Discussion section, with consideration of possible confounding by indication and selection effects.

### 3.4 Identification of lifestyle and psychosocial profiles using cluster analysis

K-means cluster analysis identified three distinct participant profiles based on age, psychological well-being, social participation, and social connection scores (Table 4). The cluster solution was statistically significant across all clustering variables (all p < .001).

**Table 4:** Profiles of elderly groups identified via K-means cluster analysis.

Variable	Cluster 0	Cluster 1	Cluster 2	F value	p
Sample size	378	410	311	—	—
Age	83.75 (6.12)	83.14 (5.94)	70.70 (4.92)	512.4	< .001
WHO-5 Well-being	8.24 (2.91)	13.85 (3.42)	10.87 (3.11)	384.2	< .001
Social Participation	40.80 (7.21)	28.04 (6.33)	40.72 (7.04)	602.7	< .001
Social Connection	42.24 (4.01)	34.23 (4.88)	42.26 (3.96)	721.6	< .001

Cluster interpretation:

Cluster 0: Older–Low well-being–High participation

Cluster 1: Older–High well-being–Low participation

Cluster 2: Younger–Moderate well-being–High participation

Cluster 0 (n = 378) consisted of older participants (M = 83.75 years) with the lowest psychological well-being scores (M = 8.24), but relatively high levels of social participation and social connection.

Cluster 1 (n = 410) included older participants (M = 83.14 years) with the highest psychological well-being scores (M = 13.85), but notably lower levels of social participation and social connection compared to other clusters.

Cluster 2 (n = 311) consisted of relatively younger participants (M = 70.70 years), with moderate well-being scores (M = 10.87) and high levels of both social participation and social connection.

These findings highlight the heterogeneity of psychosocial and lifestyle profiles among rural older adults, suggesting that individuals may experience different patterns of psychological well-being and social engagement despite similar participation in community-based lifestyle interventions.

## 4 Discussion

### 4.1 Lifestyle interventions and psychological well-being

The present study found that participation in community-based lifestyle intervention domains was associated with psychological well-being outcomes among rural older adults. These findings are consistent with a large body of literature demonstrating associations between lifestyle interventions—including physical activity, nutrition, and psychosocial engagement—and psychological well-being in older populations [[1], [2], [3]]. Such interventions are proposed to influence well-being through multiple mechanisms, including increased self-efficacy, mastery experiences, and positive cognitive reframing, which may directly influence psychological outcomes rather than merely acting as secondary consequences of improved physical health [2].

Importantly, community-based health promotion programs have been shown to produce significant improvements in psychological health, physical activity, and chronic disease self-management, particularly when delivered at the community level rather than through individual interventions [5]. Similarly, community health education programs have demonstrated significant improvements in psychological health, health promotion behaviors, and overall well-being among older adults [4]. These findings suggest that lifestyle interventions embedded within community environments may provide more comprehensive psychosocial benefits by simultaneously addressing behavioral, cognitive, and social determinants of health.

The WHO-5 well-being index used in this study is a validated and reliable measure of subjective well-being among older adults and has demonstrated strong psychometric properties across diverse populations [28]. Prior research using WHO-5 has demonstrated that well-being is influenced by multiple factors, including physical health, psychological resilience, social support, and community belonging. These multidimensional influences align with the present study’s findings, which highlight the importance of integrated lifestyle interventions in supporting psychological well-being.

Several findings in this study require careful, nuanced interpretation. Specifically, greater vision health participation hours were associated with higher well-being scores (Table 2) but also with elevated social isolation risk (Table 3), while greater hearing health participation was associated with lower well-being but reduced isolation risk. These counterintuitive patterns may reflect confounding by indication: older adults with greater sensory impairments—who tend to have higher baseline psychosocial vulnerability—may be preferentially referred to or self-select into sensory health intervention components. Under this interpretation, higher participation in these domains may serve as a proxy for greater underlying need rather than as a harmful exposure. This interpretation is also consistent with the modest variance explained by the regression models (R² = .064 for well-being; Nagelkerke R² = .118 for isolation risk), which indicates that unmeasured confounders—including baseline functional and psychosocial status—are likely important determinants of outcomes. These findings should not be interpreted as evidence that sensory health interventions cause harm. Rather, they signal the importance of accounting for pre-intervention risk profiles in future research, ideally through stratified analyses or adjustment for baseline psychosocial and functional measures, to disentangle true intervention effects from participant selection processes.

It is also important to acknowledge a key methodological assumption underlying the regression analyses. The linear modeling approach used here assumes a constant association between each additional hour of participation and well-being outcomes. However, dose–response relationships in lifestyle interventions are often non-linear, with threshold effects and potential ceiling effects at higher levels of exposure. It is plausible that marginal gains in well-being diminish beyond a certain participation intensity, or that some individuals require a minimum dosage before benefits become detectable. Future research should examine non-linear associations—for example, using spline regression or categorical dosage bands—to identify optimal participation ranges for each Green Care domain and to more accurately characterize the shape of the exposure–outcome relationship.

Furthermore, non-pharmacological interventions such as music therapy and horticultural activities have demonstrated significant improvements in psychological well-being, cognitive function, and social engagement among community-dwelling older adults. These findings reinforce the importance of integrating diverse lifestyle intervention components—including cognitive, sensory, and environmental engagement—into community-based health promotion programs.

### 4.2 Social participation, social prescribing, and social isolation

The present findings also demonstrated associations between lifestyle intervention participation and social isolation risk, which is consistent with prior research highlighting the importance of social participation as a core determinant of healthy aging [6,7]. Lifestyle interventions delivered in group-based settings provide opportunities for social interaction, peer support, and routine engagement, which contribute to improved psychological and social outcomes [29].

Social prescribing frameworks provide additional theoretical support for these findings. Social prescribing involves connecting individuals with community-based social activities to address social determinants of health and improve well-being [[8], [9]]. These interventions have demonstrated effectiveness in reducing social isolation, improving psychological well-being, and enhancing social connectedness [10]. Participation in community activities such as gardening, social groups, and volunteering has been associated with increased social engagement and improved psychological well-being [10].

Social prescribing interventions are particularly important for older adults because many health challenges in later life are driven by social and environmental factors rather than purely medical conditions [8]. By connecting individuals with community resources, social prescribing interventions can address social isolation and improve overall well-being. However, previous research has also noted variability in outcomes and emphasized the importance of long-term engagement, individualized support, and strong community infrastructure to ensure sustained benefits [[11], [12], [13]].

The present findings support the potential role of community-based lifestyle interventions as a form of social prescribing, particularly in rural settings where healthcare access may be limited. These interventions may enhance social connectedness by providing structured opportunities for interpersonal engagement and community participation.

### 4.3 Community-based interventions and healthy aging

Community-based lifestyle interventions represent a key strategy for promoting healthy aging at the population level. Prior research has demonstrated that community-based interventions improve mental well-being, vitality, and social functioning [30]. Multicomponent community interventions integrating physical, nutritional, and social components have demonstrated particularly strong effects among vulnerable older adults [31]. These interventions are effective because they address multiple determinants of health simultaneously.

Community-based health promotion programs also improve participation, adherence, and long-term sustainability by leveraging existing social networks and community infrastructure [32]. Participation in community-based health promotion activities is influenced by perceived benefits, self-efficacy, and social support [33]. Enhancing social support and providing accessible community programs may therefore improve participation and intervention effectiveness. These findings highlight the importance of community-based intervention delivery models, particularly in rural settings where healthcare access may be limited.

### 4.4 Nature-based interventions and green care

Nature-based interventions, including Green Care programs, represent an emerging and increasingly recognized approach to promoting psychological well-being and healthy aging among older adults. The findings of the present study align with a growing body of evidence demonstrating that engagement with natural environments is associated with substantial psychological and emotional benefits. Systematic reviews and empirical studies consistently indicate that participation in horticultural therapy, Green Care farming, and nature-based walking programs is associated with reduced symptoms of depression and anxiety and with improvements in subjective well-being, emotional regulation, and overall life satisfaction [[14], [15], [16],^34^]. Importantly, these associations have been observed across diverse settings, including institutional long-term care environments and tropical climates, suggesting that nature-based interventions may remain beneficial even in constrained or non-traditional environments. For example, therapeutic horticultural activities conducted within residential care settings have been associated with improved cognitive functioning and positive emotional states among older adults [35,36], highlighting the potential adaptability and broad applicability of these approaches.

The effectiveness of nature-based interventions can be understood through multiple complementary theoretical and psychosocial mechanisms. In addition to established theoretical frameworks such as Attention Restoration Theory (ART) and Stress Reduction Theory (SRT), which emphasize the restorative psychological effects of natural environments [20], social interaction has emerged as a critical mediating factor. Group-based community gardening and Green Care programs provide opportunities for shared experiences, collaborative engagement, and interpersonal connection, which contribute to reduced social isolation and enhanced social connectedness [17], [18], [19]. Through participation in meaningful nature-related activities, older adults develop peer support networks and experience increased feelings of belonging, responsibility, and social inclusion. These experiences foster psychological empowerment, improve self-efficacy, and contribute to a stronger sense of purpose and meaning in life ([14]; Yeo et al., n.d.). Such psychosocial benefits extend beyond individual-level improvements, supporting broader social integration and emotional resilience among older adults.

Community-based delivery of nature-based interventions further enhances their feasibility, accessibility, and long-term sustainability. Green social prescribing initiatives, which connect individuals to nature-based community activities through healthcare and social service systems, have gained increasing acceptance among primary care providers and are considered effective non-pharmacological interventions for older adults with multiple chronic conditions [21]. Participation in community-based Green Care programs has been associated with significant improvements in mental health outcomes, increased motivation for continued engagement, and enhanced adherence to healthy lifestyle behaviors [14], [18]. These findings highlight the importance of integrating nature-based interventions into community and primary care systems as scalable and sustainable approaches to promoting healthy aging.

Furthermore, nature-based interventions are highly consistent with broader theoretical frameworks of active aging and healthy aging. Rather than serving solely as recreational activities, Green Care interventions represent holistic, multidimensional approaches that integrate physical activity, informal lifelong learning, psychological empowerment, and social engagement [16], [19], [37]. These interventions support multiple domains of functional ability and well-being, contributing to improved quality of life and resilience among older adults. By providing opportunities for meaningful engagement with natural environments, Green Care interventions facilitate both individual-level psychological benefits and broader community-level social integration.

Taken together, the findings of the present study and the existing literature provide strong evidence supporting the integration of nature-based interventions and Green Care programs into community-based aging care systems. Expanding access to diverse nature-based activities may serve as an effective strategy for promoting psychological well-being, reducing social isolation, and enhancing overall quality of life among older adults. Future public health policies and community care models should prioritize the incorporation of Green Care and nature-based interventions to build more resilient, supportive, and sustainable health promotion systems for aging populations.

### 4.5 Intrinsic capacity, icope framework, and precision social prescribing

The World Health Organization’s Integrated Care for Older People (ICOPE) framework is a person-centred, integrated health and social care approach designed to promote healthy aging at the community level [22,23]. Rather than functioning solely as a screening instrument, ICOPE encompasses a continuum of care that spans multidimensional assessment of intrinsic capacity (IC) across core domains—including cognition, mobility, vitality, psychological function, and sensory capacity—individualized care planning, and structured linkage to community services and activities. The present study situates itself within this broader ICOPE framework by exploring its potential as a classification system for structuring community participation programs and lifestyle interventions. Importantly, while the Green Care program’s domain structure is conceptually aligned with ICOPE’s capacity domains, the present study did not implement formal ICOPE-guided referral matching; the ICOPE framework is therefore invoked as a conceptual lens rather than a description of current practice. This distinction is central to appropriately scoping the study’s contribution.

Specifically, the domain-based structure of ICOPE provides a theoretically grounded and operationally feasible framework for categorizing community-based health promotion programs and aligning them with older adults’ functional needs [24], [38]. Each intrinsic capacity domain corresponds to distinct intervention targets. For example, older adults with reduced mobility may benefit from physical activity and exercise programs, while those with psychological or cognitive vulnerabilities may benefit from social engagement, horticultural therapy, or lifelong learning programs. Integrating ICOPE into community program design allows for the systematic classification of health promotion activities—including green care, horticultural therapy, physical activity, social participation, and cultural engagement—based on their targeted functional domains. Empirical studies have shown that community-based interventions designed according to ICOPE principles have been associated with improved subjective well-being (WHO-5), psychological resilience, and overall functional ability among older adults [39,40]. These findings suggest that ICOPE-informed interventions may extend beyond recreational activities and serve as structured pathways for lifelong learning, psychological empowerment, and social integration [38], [41].

Importantly, the present study highlights the potential of ICOPE to serve as a foundational framework for precision social prescribing. Social prescribing emphasizes the referral of individuals to non-medical community interventions to improve health and well-being, particularly for individuals with psychosocial vulnerabilities [42]. However, one of the key challenges in social prescribing implementation has been the lack of standardized tools for identifying individual needs and matching individuals with appropriate interventions. The ICOPE framework addresses this gap by providing a systematic method for identifying domain-specific functional vulnerabilities through intrinsic capacity screening [[24], [25]]. Based on ICOPE assessment results, healthcare providers and community practitioners can guide older adults toward targeted interventions designed to strengthen weaker functional domains. For example, older adults identified as having psychological vulnerability may be referred to social participation or nature-based programs, while those with mobility decline may be referred to exercise-based interventions. This domain-based referral approach enables the implementation of precision social prescribing, ensuring that interventions are tailored to individual functional profiles rather than delivered through generalized, non-specific programming [39,42].

Furthermore, integrating ICOPE into community-based intervention systems strengthens the linkage between healthcare and community services, creating a more coordinated and person-centered care model. Community-based programs aligned with intrinsic capacity domains have demonstrated effectiveness in improving functional ability, reducing frailty risk, and enhancing overall well-being [24]. Additionally, early identification of intrinsic capacity decline enables preventive intervention before the onset of disability, which is consistent with the preventive and proactive philosophy of healthy aging [23], [43]. Through this integrated model, older adults transition from passive recipients of care to active participants in health promotion, social engagement, and lifelong learning.

Taken together, the findings of this study suggest that the ICOPE framework can play a dual role in community aging systems. As a person-centred, integrated care approach, ICOPE can support both the assessment of individual functional vulnerabilities and the structuring of community-based health promotion programs, thereby facilitating more targeted social prescribing. However, it should be emphasized that this study demonstrates the conceptual plausibility of such integration rather than empirically testing an ICOPE-guided referral model. Future research involving implementation studies and randomized designs is needed to evaluate whether ICOPE-guided matching of individuals to intervention domains improves outcomes beyond standard community participation. Integrating ICOPE into community care systems may ultimately strengthen the coordination between healthcare and community services, supporting person-centred, sustainable healthy aging ecosystems.

### 4.6 Cluster profiles and implications for tailored social prescribing

The three clusters identified in this study represent conceptually distinct psychosocial profiles within the program population, each with different implications for social prescribing practice. Cluster 0 (n = 378; older, low well-being, high participation and social connection) may represent individuals experiencing psychological distress despite active social engagement—a pattern that suggests participation volume alone may be insufficient to address their psychological needs. This group may benefit from more intensive psychosocial support, mental health referral, or intervention modalities specifically targeting psychological domain decline within an ICOPE framework. Cluster 1 (n = 410; older, high well-being, low participation and social connection) presents a paradox: subjective well-being is preserved despite low social engagement. This may reflect a subgroup who have achieved psychological equilibrium through intrapersonal resources such as acceptance, spirituality, or established routines, and who may not perceive a need for group-based programming. Social prescribing strategies for this group should prioritize accessible, low-barrier activities that do not require high commitment, and may benefit from motivational interviewing approaches to facilitate engagement. Cluster 2 (n = 311; younger, moderate well-being, high participation) likely represents the program’s most functionally capable and motivationally responsive subgroup, who may benefit most from diverse, higher-intensity intervention options across multiple domains. Collectively, these profiles illustrate how a one-size-fits-all program model may not optimally serve all participants, and support the case for differentiated, needs-based participation pathways. It should be noted that the K-means cluster solution is exploratory and data-driven; these profiles should not be generalized as universal typologies without replication across different populations and program contexts. Whether intervention participation patterns differ systematically by cluster could not be assessed in the present study due to the nature of exposure data, but represents an important direction for future research.

### 4.7 Policy implications: toward ICOPE-Guided precision community participation and social prescribing

The findings of the present study have important policy implications for the design and delivery of community-based social participation and health promotion programs for older adults. Cluster analysis revealed substantial heterogeneity in psychological well-being, social participation, and intrinsic capacity among older adults, which is consistent with prior research demonstrating significant variation in social vulnerability, psychological resilience, and functional health status within aging populations [26,27]. Despite this well-documented heterogeneity, current community-based participation programs are typically designed as standardized, one-size-fits-all interventions that provide uniform course content to older adults regardless of their individual intrinsic capacity, psychological status, or health risk profile. While such programs play an important role in promoting social connectedness and reducing social isolation, their ability to address domain-specific functional decline and optimize individual health outcomes remains limited.

The present study suggests that integrating the WHO Integrated Care for Older People (ICOPE) framework into community-based service systems can fundamentally transform this conventional service model. By using ICOPE as a functional health assessment tool, practitioners can identify individual intrinsic capacity profiles and functional vulnerabilities. More importantly, ICOPE can serve as a classification framework for organizing and categorizing community-based participation programs according to their targeted functional domains, such as mobility, cognition, psychological well-being, and vitality. This framework enables the alignment of older adults’ functional needs with appropriate intervention types, thereby supporting the development of heterogeneous participation pathways tailored to individual health profiles. Such an approach moves beyond generalized social participation toward individualized engagement strategies that simultaneously promote social connection and strengthen specific functional capacities.

This ICOPE-guided model also provides a practical and scalable foundation for implementing precision social prescribing. In practice, operationalizing this model in rural Taiwan would involve several key components. First, a link-worker role—fulfilled by community health workers (e.g., public health nurses), social workers attached to community care centers, or primary care nurses—would conduct brief ICOPE-aligned screenings to identify domain-specific capacity vulnerabilities. Second, assessment findings would inform structured referral pathways directing older adults toward Green Care intervention domains aligned with their identified needs (e.g., individuals with psychological domain decline referred to group-based horticultural therapy or social support activities; those with mobility decline referred to green exercise programs). Third, follow-up mechanisms would monitor participation and reassess functional status over time. Implementation in rural and resource-constrained settings would require attention to workforce capacity, transportation access, and community infrastructure, as these are well-documented barriers to social prescribing in non-urban contexts [13]. Digital health tools and telehealth-linked referral platforms may offer scalable solutions in settings with limited face-to-face service infrastructure. It is important to acknowledge that the present study did not test ICOPE-guided referral matching; accordingly, the precision social prescribing model described here represents a plausible, evidence-informed framework rather than a demonstrated practice. Community-based interventions aligned with ICOPE and social prescribing frameworks represent potentially scalable and sustainable strategies for promoting healthy aging. Future implementation studies and randomized evaluations are needed to test the feasibility and effectiveness of ICOPE-guided personalized participation models in real-world community settings.

### 4.8 Limitations

Several limitations should be considered when interpreting the findings of this study. First, the cross-sectional design limits the ability to establish causal relationships between participation in community-based lifestyle intervention domains and psychological well-being. Although significant associations were observed, it remains unclear whether participation improves well-being or whether individuals with better well-being are more likely to engage in community activities. Longitudinal and intervention studies are needed to clarify causal relationships.

Second, psychological well-being and participation were assessed using self-reported measures, which may be subject to recall and response bias. Although the WHO-5 is a validated measure of subjective well-being, it does not capture objective functional or clinical outcomes. Future studies incorporating objective health and functional indicators would provide a more comprehensive evaluation.

Third, the study focused on community-dwelling older adults in rural settings, which may limit generalizability to urban populations or institutionalized older adults. Additionally, the cluster analysis approach was exploratory and data-driven, and participation patterns may vary across different populations and contexts.

Fourth, a critical limitation concerns the absence of baseline intrinsic capacity assessments. Without pre-program ICOPE screening data, it is not possible to directly test the core hypothesis that individuals with domain-specific capacity declines benefit more from participation in aligned intervention domains compared to non-aligned domains. For example, the present study cannot determine whether individuals with baseline mobility decline who participated in Green Exercise experienced greater well-being improvements than those with mobility decline who participated only in nutrition or psychological support interventions. Directly testing such matched-intervention effects would require (1) ICOPE screening at baseline, (2) longitudinal tracking of domain-specific participation intensity, and (3) repeated well-being assessments. The present study’s contribution is therefore limited to demonstrating conceptual alignment between Green Care program structure and ICOPE domains and identifying plausible mechanisms—such as confounding by indication—that may explain observed participation patterns. Prospective studies implementing ICOPE-guided referral are essential next steps for advancing this research agenda.

Fifth, the sample’s characteristics also limit generalizability. The cohort was predominantly female (74.6%) and older-old (mean age 79.83 years), reflecting patterns of voluntary program participation rather than the broader rural older-adult population. Findings may therefore not fully apply to young-old adults (aged 65–74 years), male participants, or urban settings. Additionally, rural Taiwan’s strong agricultural traditions, existing community care infrastructure, and cultural context—including norms of filial piety and collectivism—may influence both participation motivation and well-being in ways that differ from other regions. Replication in more diverse geographic, demographic, and cultural contexts is needed before the findings can be broadly generalized.

Finally, although this study explored the potential of the ICOPE framework as a conceptual classification structure for community participation and precision social prescribing, formal ICOPE-guided intervention matching was not implemented or tested. The ICOPE framework was applied as a conceptual lens rather than a clinical protocol, and any conclusions regarding precision social prescribing should be understood as hypothesis-generating rather than empirically demonstrated. Future longitudinal and implementation studies are needed to evaluate the feasibility and effectiveness of ICOPE-based personalized participation models in community settings. Additionally, the regression models explained modest proportions of outcome variance (R² = .064 for well-being; Nagelkerke R² = .118 for social isolation risk). Although these effect sizes are modest, they are comparable to those reported in other observational studies of community-based lifestyle interventions, where social and behavioral outcomes are inherently multidetermined. The majority of residual variance is likely attributable to unmeasured factors such as baseline resilience, chronic health conditions, family support, and socioeconomic resources—variables that were not available in the present dataset. Importantly, even modest population-level improvements in psychological well-being carry meaningful public health implications given the low-cost, scalable, and low-risk nature of community-based Green Care programs. The cluster solution was exploratory and data-driven; the three participant profiles identified should not be interpreted as universal or fixed typologies, and replication across different populations and program contexts is needed before generalization.

Despite these limitations, this study provides important evidence supporting the expanded role of the ICOPE framework as both a functional assessment tool and a structural foundation for developing personalized, community-based lifestyle interventions and precision social prescribing strategies.

### 4.9 Future research directions

To translate the present hypothesis-generating findings into evidence-based practice, a phased research agenda is proposed. In Phase 1, a prospective cohort study incorporating baseline ICOPE screening would allow direct examination of whether domain-specific intrinsic capacity decline at baseline moderates the association between participation in aligned Green Care intervention domains and subsequent well-being outcomes. This phase would provide critical evidence for or against the precision-matching hypothesis central to ICOPE-guided social prescribing.

In Phase 2, a quasi-experimental implementation study could evaluate the real-world feasibility and effectiveness of structured ICOPE-guided referral pathways within community care centers. This phase would assess whether link-worker-facilitated ICOPE screening followed by domain-matched Green Care participation produces measurably better psychosocial outcomes compared to standard, non-guided community program enrollment. Process evaluation components should examine barriers and facilitators to implementation in rural and resource-constrained settings, with particular attention to workforce capacity and transportation access.

In Phase 3, if Phase 1 and 2 findings support the efficacy and feasibility of ICOPE-guided matching, a cluster-randomized trial could be conducted to provide definitive causal evidence. Community care centers would be randomly assigned to implement either ICOPE-guided precision social prescribing or standard community health promotion programming. Primary outcomes would include longitudinal changes in intrinsic capacity domain scores and psychological well-being, with secondary outcomes addressing social isolation risk, functional independence, and healthcare utilization. Together, this phased approach would progressively build the evidence base needed to translate the conceptual promise demonstrated in the present study into scalable, sustainable, and person-centred community care practice.

## 5 Conclusion

This study found that participation in community-based lifestyle interventions, including nature-based and Green Care activities, was associated with psychological well-being outcomes among rural older adults. These findings support the importance of community-based and lifestyle-oriented approaches in promoting healthy aging, particularly in settings where access to formal healthcare services may be limited. Nature-based interventions provide meaningful opportunities for social engagement, psychological restoration, and functional health promotion, suggesting their potential value as scalable and sustainable components of community aging care systems.

Importantly, this study contributes to discussions around the expanded application of the World Health Organization’s Integrated Care for Older People (ICOPE) framework. As a person-centred, integrated care approach encompassing assessment, care planning, and community linkage, ICOPE offers a promising conceptual structure for organizing community participation programs and informing precision social prescribing—though the present study did not directly test ICOPE-guided referral matching and these applications should be understood as plausible and hypothesis-generating. By conceptually aligning intrinsic capacity domains with targeted lifestyle and Green Care interventions, the ICOPE framework may support more personalized, needs-based participation pathways that address the heterogeneity of aging populations.

Overall, integrating ICOPE-guided assessment with community-based lifestyle and nature-based interventions represents a promising strategy for enhancing psychological well-being, promoting functional ability, and advancing precision health promotion for older adults. Future community care models and public health policies should prioritize the integration of ICOPE and Green Care approaches to support sustainable and person-centered healthy aging.

## Ethical statement

**Studies on Human and/or Animal Statement.** This study involved human participants only; no animal subjects were involved. All procedures performed in studies involving human participants were in accordance with the ethical standards of the institutional research committee and with the 1964 Declaration of Helsinki and its later amendments, or comparable ethical standards.

**Informed Consent.** Written informed consent was obtained from all individual participants included in the study prior to data collection. Participants were informed of the study’s purpose, procedures, voluntary nature of participation, confidentiality safeguards, and their right to withdraw at any time without consequence.

**Ethical Approval and Registration.** This study was reviewed and approved by the Institutional Review Board of the National Health Research Institutes (NHRI), Taiwan (IRB Approval No EC1130907-E). All study procedures complied with the ethical standards of the responsible institutional and national committees on human experimentation.

## Data statement

The de-identified datasets generated and analyzed during the current study are not publicly available because they contain sensitive information about community-dwelling older adults collected through the Rural Community Green Care Program, and data-sharing restrictions are imposed by the funding agency and the ethics approval conditions. The data are, however, available from the corresponding author upon reasonable request and subject to approval from the Institutional Review Board of the National Health Research Institutes, Taiwan.

## IRB

This study was reviewed and approved by the Institutional Review Board of the National Health Research Institutes (NHRI), Taiwan (IRB No EC1130907-E). All procedures involving human participants followed established ethical guidelines for the protection of autonomy, privacy, and voluntary participation. Informed consent was obtained from all participants prior to data collection. Participation was voluntary, and respondents could decline or withdraw at any time without penalty. No identifying information was included in the analytic dataset, and all confidentiality safeguards were strictly maintained.

## Declaration of generative AI and AI-assisted technologies in the writing process

During the preparation of this work, the author(s) used generative AI tools solely to assist with language editing and readability improvement of the manuscript. No AI tool was used to generate, analyze, or interpret data, or to produce figures, images, or other artwork. After using these tools, the author(s) carefully reviewed and edited the content as needed and take(s) full responsibility for the content of the publication.

## CRediT authorship contribution statement

**Kai-Lin Liang:** Writing – review & editing, Writing – original draft, Validation, Resources, Project administration, Methodology, Investigation, Funding acquisition, Formal analysis, Conceptualization. **Yi-Chun Hung:** Software, Formal analysis, Data curation. **Chia-Chen Hung:** Resources, Data curation. **Chih-Ying Lin:** Resources, Data curation.

## Declaration of competing interest

The authors declare that they have no known competing financial interests or personal relationships that could have appeared to influence the work reported in this paper.

## Data Availability

The data that support the findings of this study are available from the corresponding author upon reasonable request. Due to participant privacy and institutional regulations, data cannot be made publicly available.

## Appendix Supplementary materials

Image, application 1
