# Supplementary material for: Community-based lifestyle interventions and psychological well-being among rural older adults: Evidence from Taiwan’s green care program
Source: JAR Life. 2026 May 28;15:100074. doi: 10.1016/j.jarlif.2026.100074 (PMC13235368; doi:10.1016/j.jarlif.2026.100074)
Supplement: Supplementary file 1 [file mmc1.docx]

**Supplementary**

Figure 1. Participant Flow Diagram


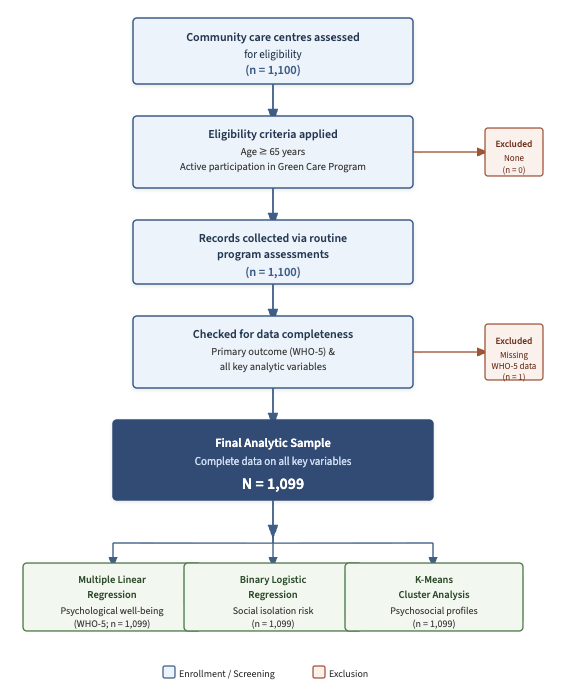


*Note. WHO-5 = World Health Organization Five Well-Being Index. Missing data were not imputed; analyses were conducted on participants with complete data across all key analytic variables.*
